# Supplementary material for: Neuroprotection following FLASH-RT may be mediated by sustained glutamate receptor AMPAR activation in CA3 neurons
Source: bioRxiv. 2026 May 19:2026.05.15.725423. Preprint. [Version 1] doi: 10.64898/2026.05.15.725423 (PMC13228234; doi:10.64898/2026.05.15.725423)
Supplement: Supplement 1 [file NIHPP2026.05.15.725423v1-supplement-1.pdf]

bioRxiv preprint doi: https://doi.org/10.64898/2026.05.15.725423; this version posted May 19, 2026. The copyright holder for this preprint (which was not certified by peer review) is the author/funder, who has granted bioRxiv a license to display the preprint in perpetuity. It is made available under a CC-BY 4.0 International license.

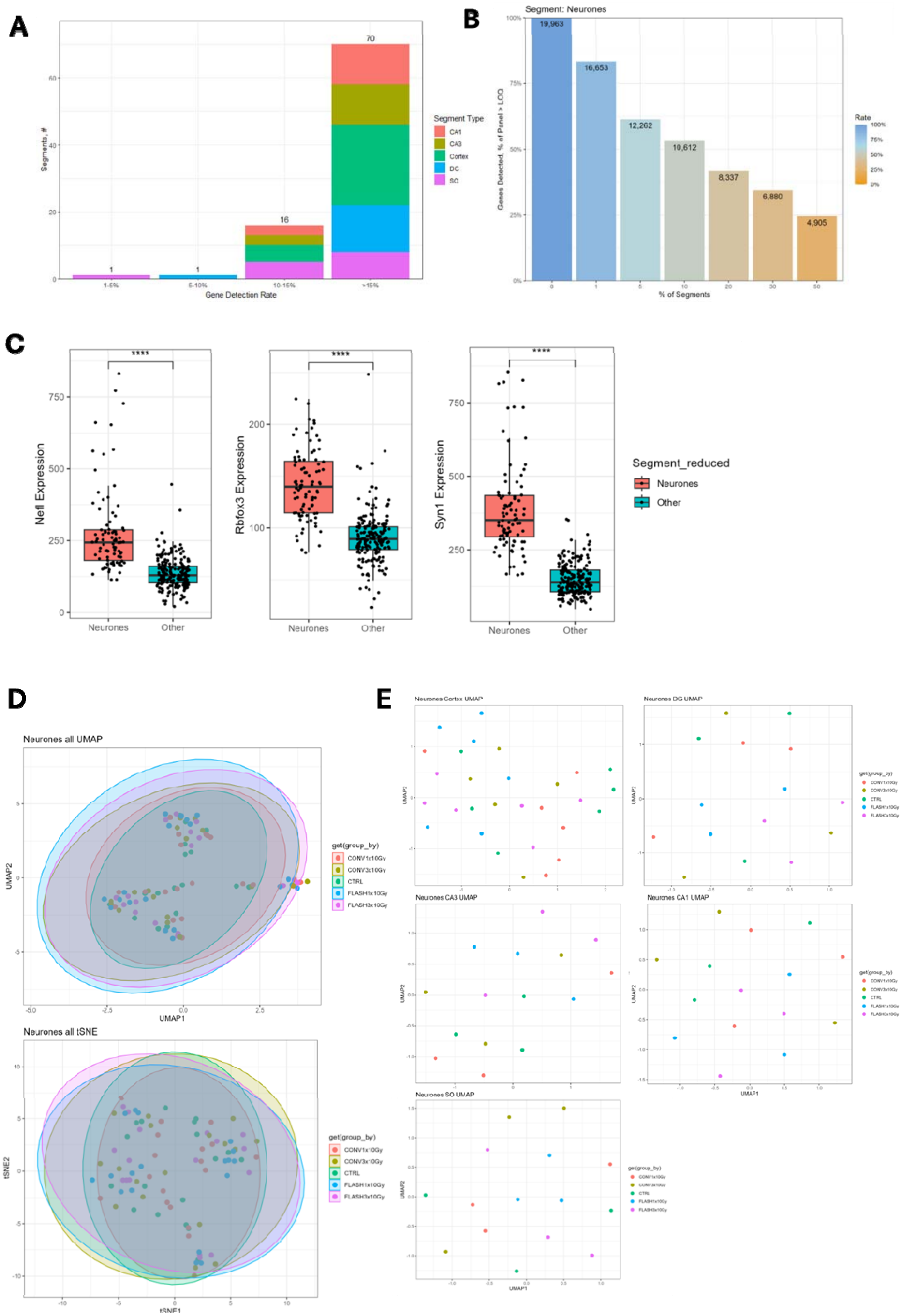

Fig. supp 1: Confirmation of robust gene detection rate within segments and confirmation of cell type specificity. (A) Histogram of number of ROI (segments) for increasing gene detection rate thresholds colored per subregions. (B) Percentage of genes detected i.e. above limit of quantification (LOQ). (C) Expression of specific markers of neurons across different cell types (as identified with immunostainings). P-values computed using a Mann-Whitney test. One, two, three and four stars respectively representing p-values smaller

bioRxiv preprint doi: https://doi.org/10.64898/2026.05.15.725423; this version posted May 19, 2026. The copyright holder for this preprint (which was not certified by peer review) is the author/funder, who has granted bioRxiv a license to display the preprint in perpetuity. It is made available under a CC-BY 4.0 International license.

than 0.05, 0.01, 0.001, 0.0001. (D) UMAP and tSNE colored per irradiation conditions for all regions (all) or (E) individual regions considered (Cortex, DG, CA3, CA1, SO). Related to STAR methods and Figure 1.

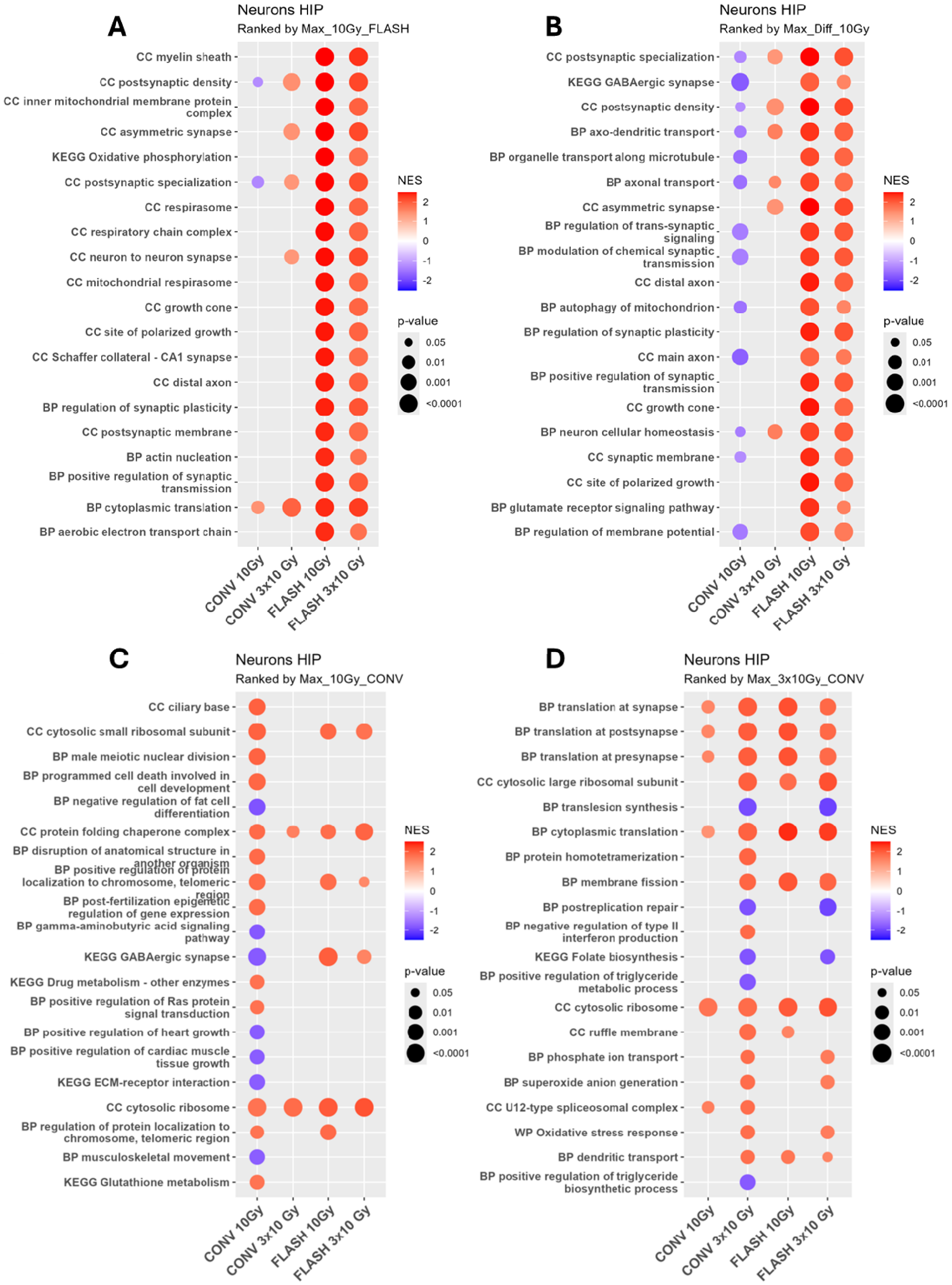

Fig. supp 2: CONV presents dose dependent imprint in the hippocampus while FLASH seems to induce a dose agnostic response. (A) Output of GSEA on Hippocampus signature,

bioRxiv preprint doi: https://doi.org/10.64898/2026.05.15.725423; this version posted May 19, 2026. The copyright holder for this preprint (which was not certified by peer review) is the author/funder, who has granted bioRxiv a license to display the preprint in perpetuity. It is made available under a CC-BY 4.0 International license.

pathways are ranked according to the maximum absolute NES of FLASH 10 Gy (B) the maximum absolute difference in NES between CONV 10 Gy and FLASH 10 Gy, (C) maximum absolute NES of CONV 10 Gy (D) or maximum absolute NES of CONV 3x10 Gy. Normalized enrichment score (NES) as the color and p-value as the dot-size.. Related to Figure 2.

bioRxiv preprint doi: https://doi.org/10.64898/2026.05.15.725423; this version posted May 19, 2026. The copyright holder for this preprint (which was not certified by peer review) is the author/funder, who has granted bioRxiv a license to display the preprint in perpetuity. It is made available under a CC-BY 4.0 International license.

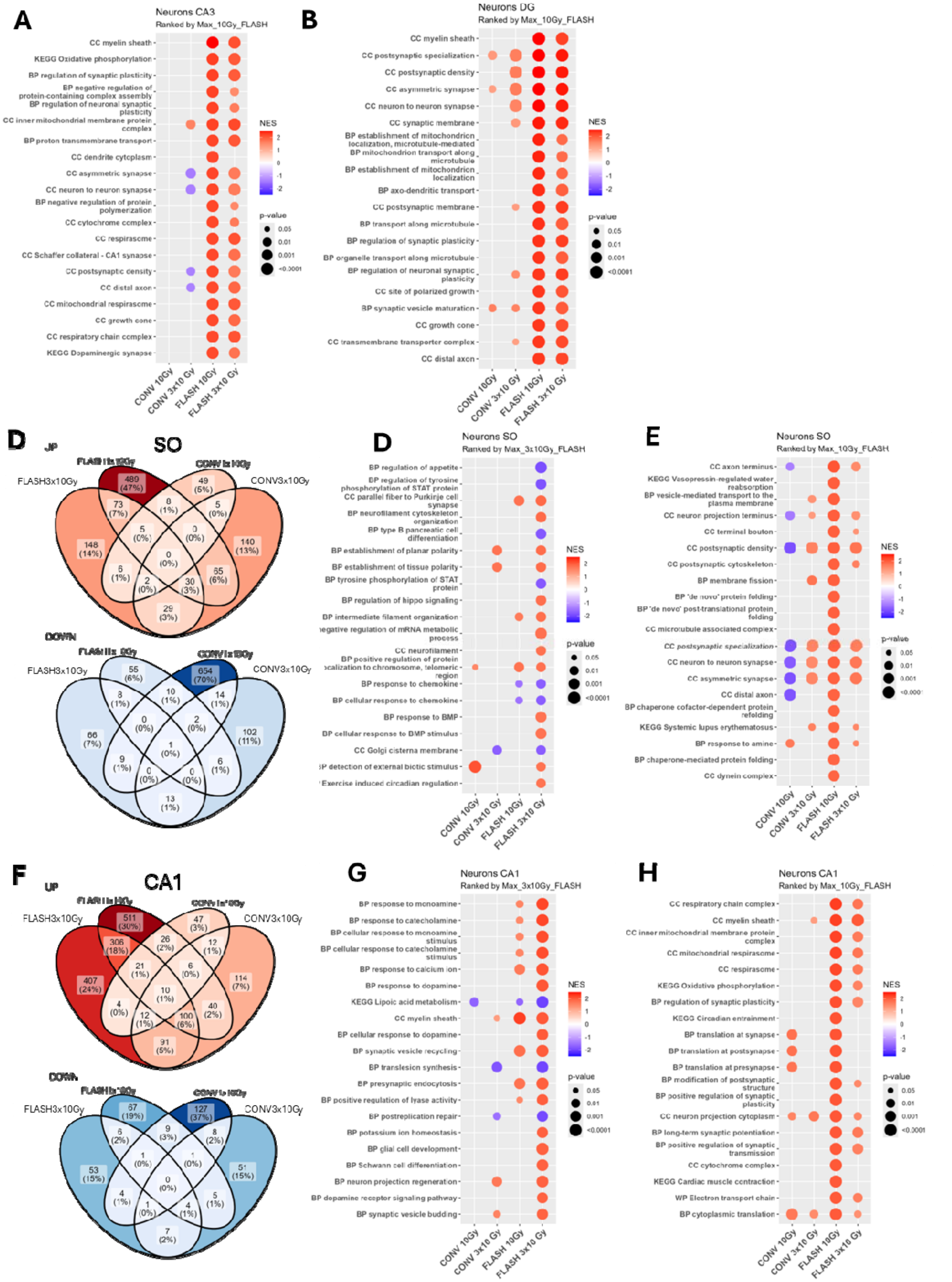

Fig. supp 3: While in the CA3 and the DG FLASH induces a dose agnostic response, in the SO and in the CA1 the imprint always depends on the dose. (A) Output of GSEA signature, pathways are ranked according to the maximum absolute NES of FLASH 10 Gy for the CA3 (B) or the DG. (C) Venn Diagrams of Up (NES > 0, p-value < 0.05) and Down (NES < 0, p-value < 0.05) regulated pathways in the SO. (D) Output of GSEA on SO signature, pathways are ranked according to the maximum absolute NES of FLASH 3x10 Gy (E) or max

bioRxiv preprint doi: https://doi.org/10.64898/2026.05.15.725423; this version posted May 19, 2026. The copyright holder for this preprint (which was not certified by peer review) is the author/funder, who has granted bioRxiv a license to display the preprint in perpetuity. It is made available under a CC-BY 4.0 International license.

absolute NES of FLASH 10 Gy. (F) Venn Diagrams of Up (NES > 0, p-value < 0.05) and Down (NES < 0, p-value < 0.05) regulated pathways in the CA1. (G) Output of GSEA on CA1 signature, pathways are ranked according to the maximum absolute NES of FLASH 3x10 Gy (H) or max absolute NES of FLASH 10 Gy. Related to Figure 3.

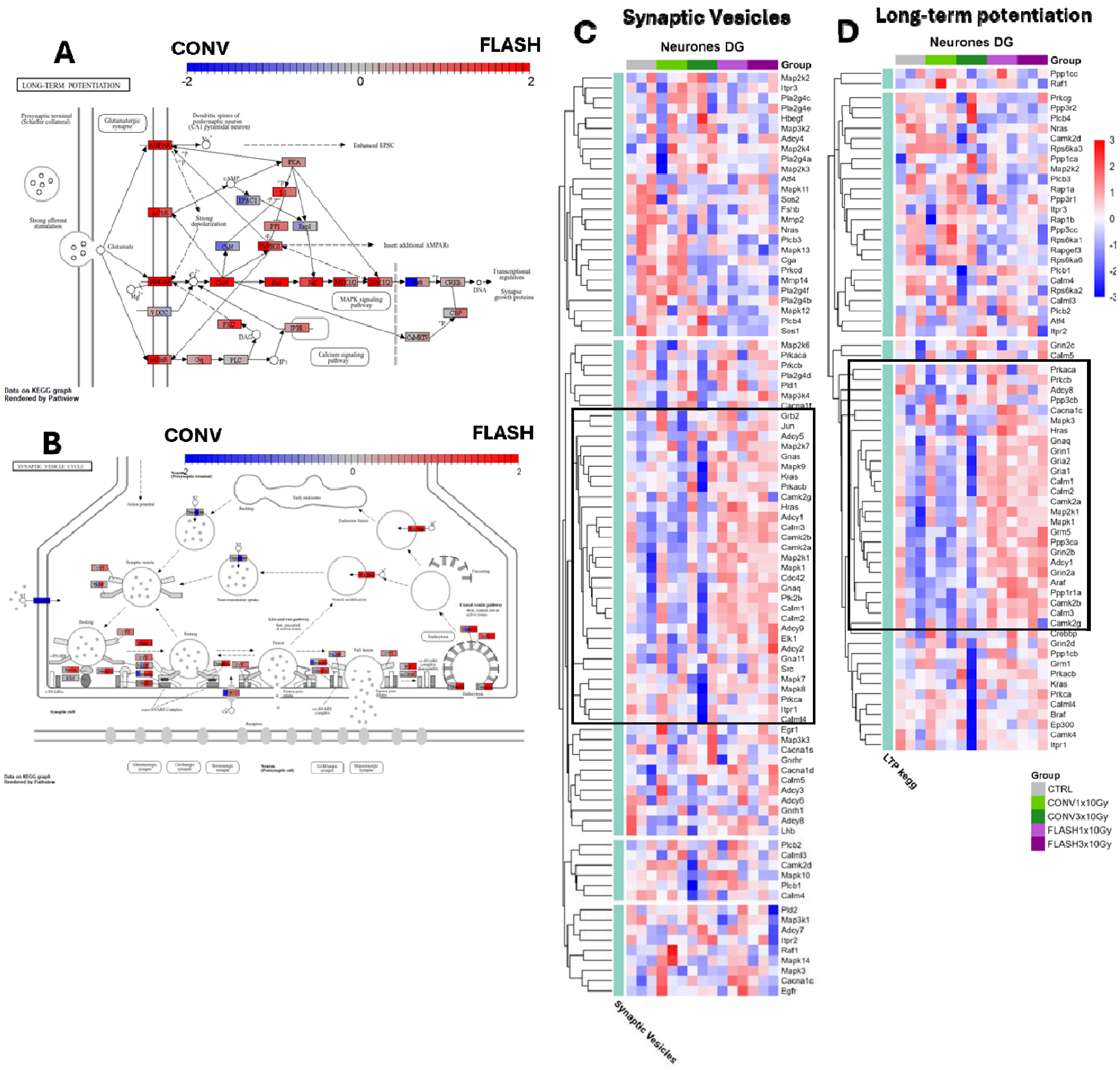

Fig. supp 4: Pathway elements and specific genes show a similar pattern in the DG as in the CA3 for long term potentiation and synaptic vesicle cycle. (A) Pathways diagrams made using *Pathview* for KEGG long-term potentiation (B) and synaptic vesicle cycle based on DG imprint. Color is - an arbitrary scale of enrichment from the highest expression in CONV (-2) to the highest expression in FLASH (+2). Each element of the pathway is split across center for CONV/FLASH comparison at 10 Gy (left) and at 3x10 Gy (right). (C) Gene regulation heatmap showing for neurons of DG, with black squares drawn arbitrarily for KEGG synaptic vesicles (D) and long-term potentiation.Related to Figure 4 and 5.

bioRxiv preprint doi: https://doi.org/10.64898/2026.05.15.725423; this version posted May 19, 2026. The copyright holder for this preprint (which was not certified by peer review) is the author/funder, who has granted bioRxiv a license to display the preprint in perpetuity. It is made available under a CC-BY 4.0 International license.

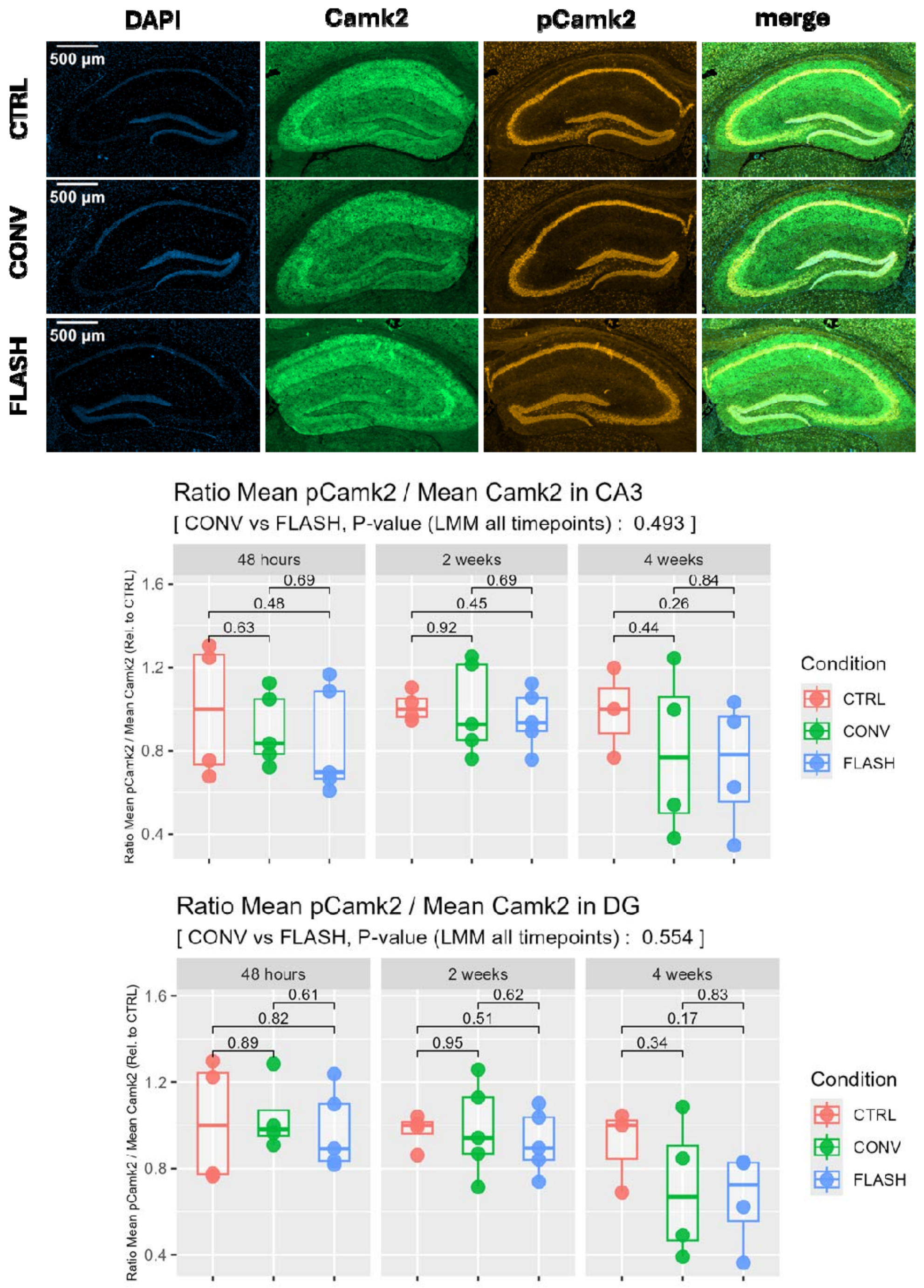

Fig. supp 5: Phosphorylated Camk2 ratio to Camk2 shows steady levels 48h, 2 weeks and 4 weeks after irradiation. Immunofluorescent staining of hippocampus of control (CTRL), FLASH 10 Gy and CONV 10 Gy irradiated mice. Staining of phosphorylated *Camk2* (p*Camk2*), *Camk2* and nuclei (DAPI). Representative images at the top and quantification across DG or CA3 at the bottom. Each point represents a different mouse and is the mean of technical replicates (n=3-6). Technical outliers were excluded based on Tukey's fences method. Pair comparison is obtained from a t-test and assessment over all timepoints was done using a LMM with mouse number and timepoint as random intercepts.. Related to Figure 5.

bioRxiv preprint doi: https://doi.org/10.64898/2026.05.15.725423; this version posted May 19, 2026. The copyright holder for this preprint (which was not certified by peer review) is the author/funder, who has granted bioRxiv a license to display the preprint in perpetuity. It is made available under a CC-BY 4.0 International license.

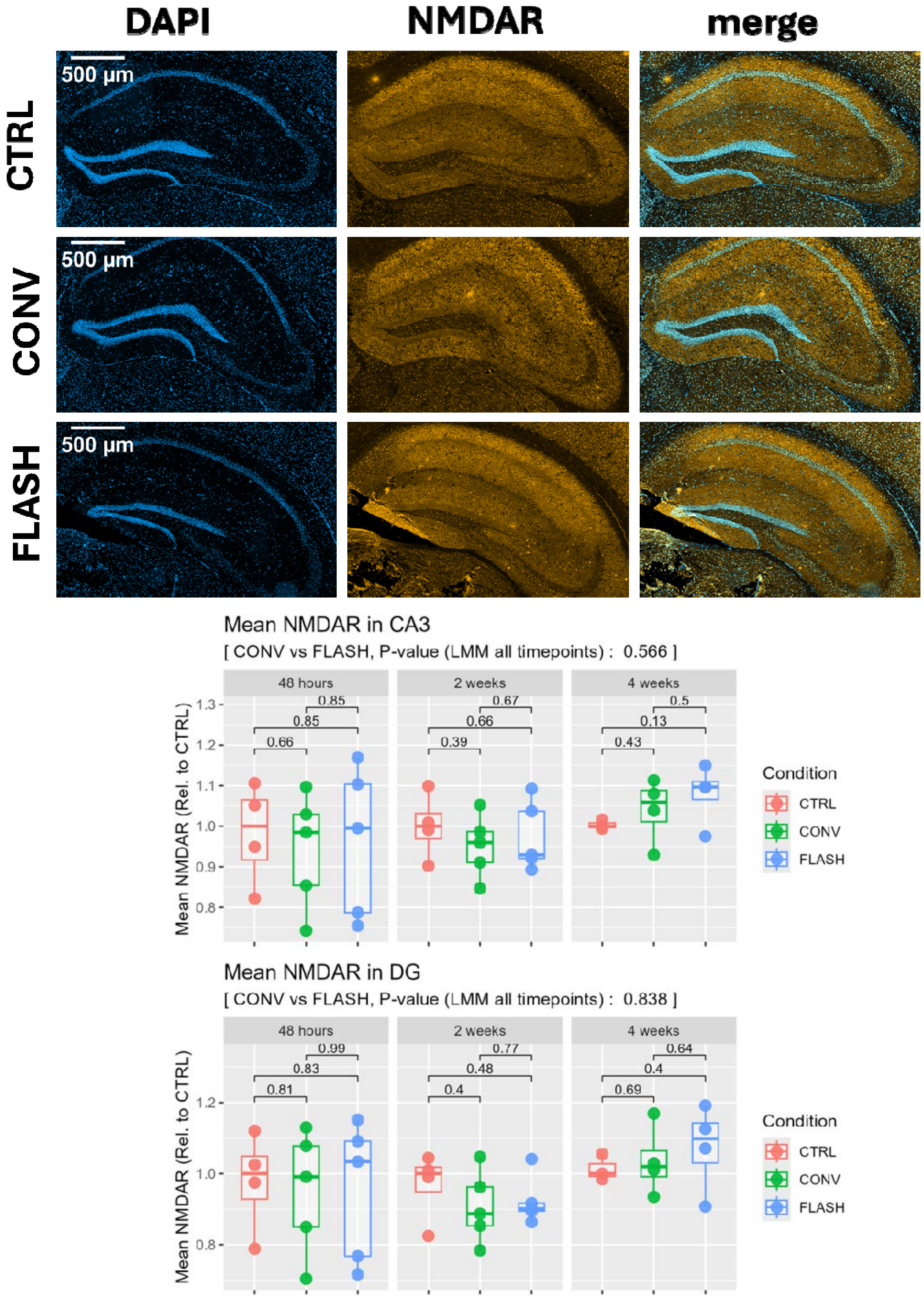

Fig. supp 6: NMDAR are maintained at constant level 48h, 2 weeks and 4 weeks after irradiation. Immunofluorescent staining of hippocampus of control (CTRL), FLASH 10 Gy and CONV 10 Gy irradiated mice. Staining of *Grin1* (NMDAR) and nuclei (DAPI). Representative images at the top and quantification across DG or CA3 at the bottom. Each point represents a different mouse and is the mean of technical replicates (n=3-6). Technical outliers were excluded based on Tukey's fences method. Pair comparison is obtained from a t-test and

bioRxiv preprint doi: https://doi.org/10.64898/2026.05.15.725423; this version posted May 19, 2026. The copyright holder for this preprint (which was not certified by peer review) is the author/funder, who has granted bioRxiv a license to display the preprint in perpetuity. It is made available under a CC-BY 4.0 International license.

assessment over all timepoints was done using a LMM with mouse number and timepoint as random intercepts.Related to Figure 6.

bioRxiv preprint doi: https://doi.org/10.64898/2026.05.15.725423; this version posted May 19, 2026. The copyright holder for this preprint (which was not certified by peer review) is the author/funder, who has granted bioRxiv a license to display the preprint in perpetuity. It is made available under a CC-BY 4.0 International license.

Supplementary table 1 : *DSP Nanostring GeoMX quality check parameters used*

QC segments	
Minimum number of reads	1000
Minimum % of reads trimmed	80
Minimum % of reads stitched	80
Minimum % of reads aligned	75
Minimum sequencing saturation	50
Minimum negative control counts	1
Minimum # of nuclei estimated	20
Minimum segment area	1000
QC genes Grubb test	
minimum Probe Ratio	0.1
percent Fail Grubbs	20
